# A Novel Nonsense Mutation of* POU4F3* Gene Causes Autosomal Dominant Hearing Loss

**DOI:** 10.1155/2016/1512831

**Published:** 2016-11-24

**Authors:** Chi Zhang, Mingming Wang, Yun Xiao, Fengguo Zhang, Yicui Zhou, Jianfeng Li, Qingyin Zheng, Xiaohui Bai, Haibo Wang

**Affiliations:** ^1^Department of Otorhinolaryngology Head and Neck Surgery, Shandong Provincial Hospital Affiliated to Shandong University, Jinan, China; ^2^Shandong Provincial Key Laboratory of Otology, Jinan, China; ^3^Department of Otolaryngology-Head & Neck Surgery, Case Western Reserve University, Cleveland, OH, USA

## Abstract

*POU4F3* gene encodes a transcription factor which plays an essential role in the maturation and maintenance of hair cells in cochlea and vestibular system. Several mutations of* POU4F3* have been reported to cause autosomal dominant nonsyndromic hearing loss in recent years. In this study, we describe a pathogenic nonsense mutation located in* POU4F3* in a four-generation Chinese family. Target region capture sequencing was performed to search for the candidate mutations from 81 genes related to nonsyndromic hearing loss in this family. A novel nonsense mutation of* POU4F3*, c.337C>T (p. Gln113^⁎^), was identified in a Chinese family characterized by late-onset progressive nonsyndromic hearing loss. The novel mutation cosegregated with hearing loss in this family and was absent in 200 ethnicity-matched controls. The mutation led to a stop codon and thus a truncated protein with no functional domains remained. Transient transfection and immunofluorescence assay revealed that the subcellular localization of the truncated protein differed markedly from normal protein, which could be the underlying reason for complete loss of its normal function. Here, we report the first nonsense mutation of* POU4F3* associated with progressive hearing loss and explored the possible underlying mechanism. Routine examination of* POU4F3* is necessary for the genetic diagnosis of hereditary hearing loss in the future.

## 1. Introduction

Hearing loss is one of the most common sensory disorders in human. Genetic factors account for about 50% of these cases. Nonsyndromic hearing loss has four hereditary patterns: autosomal dominant, autosomal recessive, X-linked, and mitochondrial. Although hundreds of genes have been reported to be associated with nonsyndromic hearing loss, GJB2, SLC26A4, and mtDNA12SrRNA are the major contributors. Sometimes, one deafness gene can exhibit both autosomal dominant and recessive patterns in different mutations, such as* WFS1* [1–3]. To date, there have been 67 loci mapped and related to autosomal dominant nonsyndromic hearing loss (ADNSHL), but only 33 corresponding genes have been identified (http://hereditaryhearingloss.org/). A large proportion of sensorineural hearing loss remains genetically unexplained. The traditional Sanger sequencing method is highly expensive and time-consuming in identifying the pathogenic variants when there are hundreds of candidate genes. In contrast, next-generation sequencing can overcome these shortcomings through its ability to perform parallel sequencing of billions of nucleotides at a low cost and high speed. It has been proven as a powerful tool in identification of novel mutations and genes associated with hereditary hearing loss in recent years.

POU class 4 transcription factor 3* (POU4F3)*, also known as* BRN3C*, is a POU-domain transcription factor exclusively expressed in both nascent and adult hair cells of cochlear and vestibular system in the inner ear [4, 5]. Brn-3c knockout mice were used to study its function in vivo. The homozygous mice of targeted deletion of* POU4F3*, Brn-3c^−/−^, manifested severe defects in hearing and balance, and they showed a rapid and progressive loss of hair cells during late gestation and early postnatal period. In contrast, the heterozygous littermates Brn-3c^+/−^ mice represented normal behaviors. Histological examinations revealed that hair cells were totally absent in the auditory and vestibular systems of Brn-3c^−/−^ adult mice. Loss of hair cells also resulted in a large decrease in the number of neurons and myelinated fibers in the spiral ganglion [6]. Other studies revealed that* POU4F3* was expressed in postmitotic cells committed to hair cell phenotype but not in mitotic progenitors [7] and the expression level of* POU4F3* kept high in both inner and outer hair cells till adulthood in mice [8], which meant* POU4F3* was essential for the maturation and maintenance, but not the fate determination of hair cells. The vital role of* POU4F3* in the development of hair cells indicated that it might be related with some kind of hereditary hearing loss.

The search for pathogenic mutations involved in hereditary hearing loss never ceases. Mutation of* POU4F3* was confirmed to be a causative factor of autosomal dominant nonsyndromic deafness 15 (DFNA15). Thus far, several* POU4F3* mutations were involved in* DFNA15* and mapped to 5q31-33 [9–16]. The main clinical manifestation is bilateral, late-onset, progressive sensorineural hearing loss affecting all frequencies [10, 12–15]. Pauw et al. reported a mean progression rate of 0.8–1.4 dB/year [17]. Vestibular impairments in some patients were also reported in previous studies, but the incidence was low and the symptoms were quite mild and easy to be neglected [17, 18].

In this study, we reported a Chinese family suffering from ADNSHL. All affected members experienced a late-onset progressive hearing loss. A new nonsense mutation in* POU4F3*, c.337C>T (p. Gln113^*∗*^), was identified to be the causative factor using the method of target region capture sequencing.

## 2. Materials and Methods

### 2.1. Subjects and Clinical Examinations

**Figure 1:**
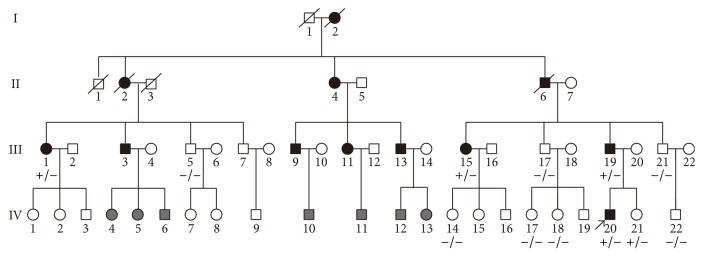
Pedigree of the Chinese family suffering from autosomal dominant hearing loss. Black squares and circles represent members with symptoms of DFNA15. Grey squares and circles represent members with unavailable status. Genotypes are marked below each member (+ means the mutation exists). Arrow shows the proband.

A four-generation Chinese family suffering from hereditary hearing loss was reported here. All 12 patients in this family had a putative autosomal dominant pattern of inheritance according to the participating patient statements. Because of some objective reasons and out of the patients' privacy, we were not able to contact and examine all the members in this family. Only 4 members with impaired hearing (III-1, III-15, III-19, and IV-20) and 8 members with normal hearing (III-5, III-17, III-21, IV-14, IV-17, IV-18, IV-21, and IV-22) participated in our research (Figure 1). They all received clinical examinations in Department of Otorhinolaryngology Head and Neck Surgery, Shandong Provincial Hospital Affiliated to Shandong University. The medical history was obtained from all participants. After physical and otoscopic examinations, all the subjects received auditory tests including pure tone audiometry (PTA), tinnitus examination, acoustic immittance, auditory brainstem response, and distortion product otoacoustic emission according to standard protocols. Vestibular bithermal caloric test and evoked myogenic potentials were performed to the proband (IV-20) due to his complaint of occasional vertigo. Other syndromic or systematic diseases which can influence hearing and past history of ototoxic medication were excluded. Degrees of hearing loss were determined according to the guidelines of American Speech-Language-Hearing Association [19]. Individual was considered affected if PTA thresholds of most frequencies were higher than the 95 percentile thresholds of presbycusis according to the method of ISO 7029-2000 [20]. Before this study, all participants provided written informed consents according to the protocol, which was approved by the ethics committee of the Institutional Review Board of the Shandong Provincial Hospital Affiliated to Shandong University.

### 2.2. Targeted Next-Generation Sequencing of Deafness Gene

In order to identify the pathogenic mutation underlying the hearing loss in this family, the genomic DNA was extracted from peripheral blood of all the subjects using DNA extraction kit (Axygen, USA). Target region capture sequencing was employed to screen possible mutations of 81 genes (see S1 Table in Supplementary Material available online at http://dx.doi.org/10.1155/2016/1512831) related to nonsyndromic hearing loss in the genome of the proband. This work was done by BGI (Beijing Genomics Institute, Shenzhen, China) using a standardized next-generation capture sequencing platform. This method can cover all exons and nearby ±10 base pairs of introns of the 81 candidate genes. Data analysis was conducted according to the analysis process for next-generation sequencing, BGIv0.1.0. Reads were aligned to the human reference genome UCSC hg19 Feb.2009 by BWA 0.6.2-r126 software. Mutation detection software was GATK. dbSNP (snp137) was used as a reference for recorded SNPs. The databases including 1000 genome database (phase I), HapMap database (combined data from phases II and III), and own databases of BGI (BGI-DB, HGVD) were used as references to investigate the novelty and possible pathogenicity of the variations detected in the sequencing approach. Guideline of American College of Medical Genetics and Genomics was used as the reference of data interpretation [21].

### 2.3. Mutation Detection by Sanger Sequencing on Genomic DNA

Sanger sequencing was performed in all the family members participating in our research and 200 ethnicity-matched control subjects. The primers used to amplify the exons and intron-exon boundaries by polymerase chain reaction were as follows: (1) forward 5′-GCAGGCTGCTTGTAAGATGAG-3′ and reverse 5′-AGACAGCGGCGATTGTTC-3′; (2) forward 5′-CTCGGTTGCTTGAAAATGTG-3′ and reverse 5′-GGGGATCTTGAGATTAGCC-3′; (3) forward 5′-AGCTGGAAGCCTTCGCC-3′ and reverse 5′-GGAAAGTCTGTGGCTTCGG-3′. The first pair of primers was for the amplification of exon 1, while the other two were for exon 2. Sequencing reactions were performed by BGI (Beijing Genomics Institute, Shenzhen, China). Data was analyzed using Lasergene-SeqMan software. The sequences were compared with the sequence of* POU4F3* gene (GenBank Accession number NM_002700) and corresponding protein sequence (NP_002691.1).

### 2.4. Bioinformatics Analysis

Mutation Taster was used to predict the possible pathogenic effect of the candidate mutation (http://www.mutationtaster.org/) [22]. Three-dimensional (3D) modeling of the human wild-type and mutant* POU4F3* protein was carried out using I-TASSER, an automated homology modeling program (http://zhanglab.ccmb.med.umich.edu/). The wild-type POU4F3 protein includes 338 amino acids (NP_002691.1) and the mutant protein includes 112 amino acids. Data obtained from the homology models were visualized using Swiss-Pdb Viewer 4.1 software.

### 2.5. Cell Culture

HEK293 cells were originally stored in Shandong Provincial Key Laboratory of Otology and then cultured in MEM (Gibco, USA) containing 10% FBS (Gibco, USA) in a sterile environment with 5% CO_2_ at 37°C. HEI-OC1 auditory cells, which were given by Dr. Federico Kalinec (University of California, Los Angeles) as a present, were cultured in DMEM (Gibco, USA) containing 10% FBS in a sterile environment with 10% CO_2_ at 33°C [23].

### 2.6. Plasmid Construction

The vector containing human* POU4F3* cDNA was purchased from Cusabio Biotech (Wuhan, China). Sanger sequencing of this cDNA clone confirmed that it is totally consistent with that of* POU4F3* cDNA (accession number: BC112207). We then used this to generate the wild-type expression plasmid. Primers used to amplify the cDNA region were 5′-ATGCAGGATCCATGATGGCCATGAACTCCAAGCAGCCTTTCG-3′ and 5′-ACGCAGAATTCGTGGACAGCCGAATACTTCA-3′. After digestion by restriction enzymes BamH I and EcoR I, the amplified PCR products were then subcloned into the expression vector, pCMV-Tag 2B (Agilent Technologies, USA). To construct the mutant expression vector, we used the QuikChange site-directed mutagenesis kit (Stratagene, USA) to introduce the mutation (c.337C>T) which we identified from targeted next-generation sequencing into the wild-type vector following the manufacturer's protocol.

### 2.7. Transient Transfection and Immunofluorescence Analysis

HEK 293 and HEI-OC1 cells were cultured on glass coverslips in 24-well plates with the densities of 25 × 10^4^/well and 10 × 10^4^/well, respectively, and were then transfected with either wild-type or mutant expression plasmid using Lipofectamine® 3000 transfection reagent (Invitrogen, USA). Immunofluorescence analysis was performed after 72-hour transfection. Cells were fixed in 4% paraformaldehyde for 15 min, permeabilized in PBS containing 0.3% Triton X-100 for 10 min and then blocked in PBS containing 10% donkey serum for 1 h at 37°C in a humid atmosphere. Subsequently, the cells were incubated for 12–14 h at 4°C with primary anti-FLAG antibody (Ca# F1804, Sigma, USA) at a concentration of 1 : 800 diluted and then stained with secondary goat-anti-mouse antibody (Sigma, USA) and DAPI for 1 h at a concentration of 1 : 1000. Finally, the cells were visualized under confocal microscope for image acquisition.

## 3. Results

### 3.1. Clinical Manifestations

**Figure 2:**
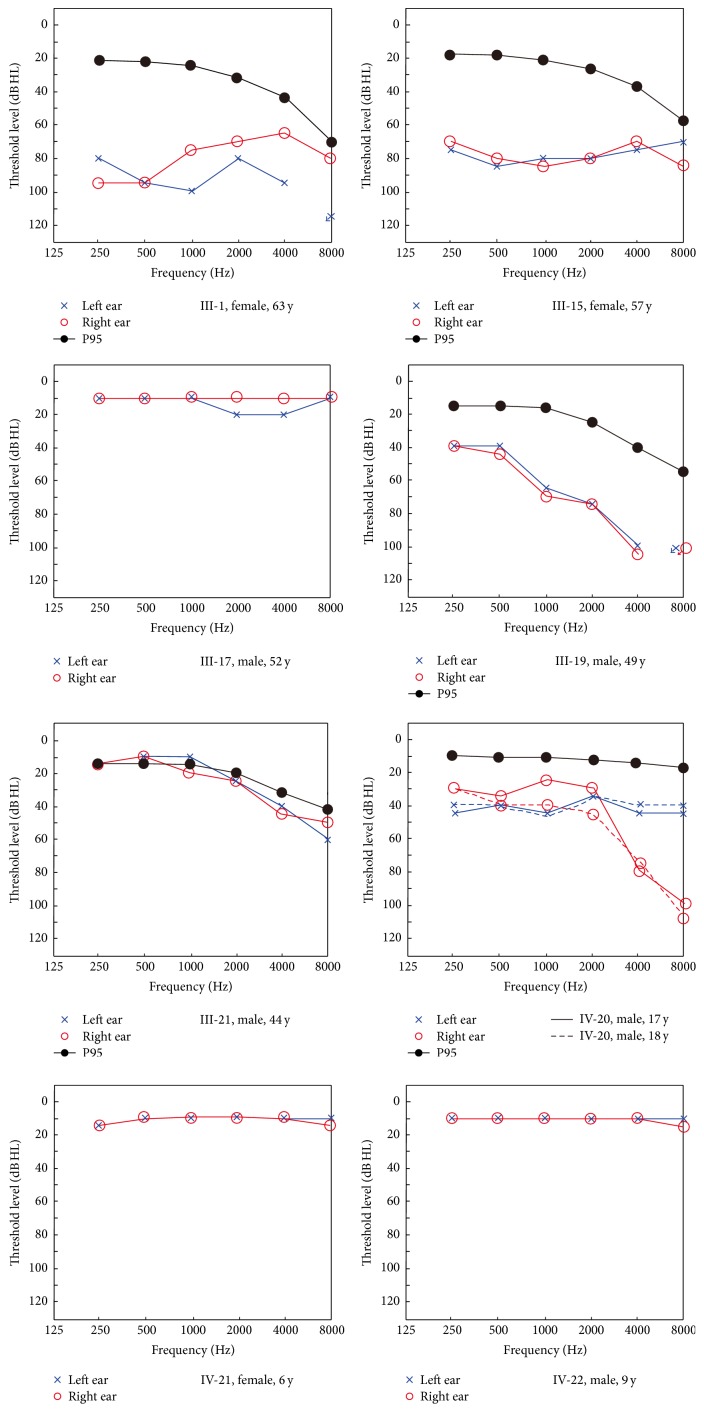
Audiograms of some members participating in our study in the Chinese family. Blue crosses and red circles represent the air conduction hearing threshold levels of left and right ears, respectively. For IV-20, hearing levels of the age of 17 and 18 years are shown in full and broken lines, respectively. Black lines with solid circles represent the 95 percentile air conduction hearing threshold levels of presbycusis with a certain gender and age according to the algorithm of ISO 7029:2000. Pedigree number, gender, and age are shown below the audiogram of each individual. “IV-20, male, 17 y” and “IV-20, male, 18 y” in the keys refer to both red and blue colors.

A four-generation Chinese family suffered from hereditary progressive hearing loss with an autosomal dominant pattern. The pedigree of this Chinese family was drawn in Figure 1 according to the statements of participants. Totally, there were 12 members suffering from similar symptoms of hearing loss and tinnitus in this family, with 9 of them still alive at the time of investigation. But out of respect for the patients' privacy and some other objective reasons, we only got 12 members to participate in our study. All the members with symptoms in this family represented bilateral late-onset progressive hearing loss, but the onset age (range, 14–40 y) varied a lot from one another. PTA results showed moderate to severe hearing loss in these patients. The audiometric configurations were flat to downsloping (Figure 2). Four of the 12 participating members including III-1, III-15, III-19, and IV-20 were assumed to be affected after PTA test and comparison with 95th percentile thresholds of presbycusis.

The proband (IV-20) developed bilateral hearing loss at the age of 14 and after then, the hearing loss became more and more severe. So he received PTA in our hospital at 17 and 18 years old, respectively. The results showed moderate sensorineural hearing loss of both ears (shown in Figure 2) and an obvious decrease of the right ear in 1 and 2 kHz was observed when comparing these two audiograms. Usually, the configurations of both ears were the same or similar, but the proband showed different configurations on each side of ears.

Tinnitus was a common symptom among these patients. Tinnitus examination revealed a 3 kHz binaural consistent tinnitus in IV-20 (left: 51 dB HL; right: 85 dB HL) and a 6 kHz binaural tinnitus in III-19 (left: 103 dB HL; right: 104 dB HL). Speech recognition scores (SRS) of IV-20 were 88% in left and 80% in right. SRS of III-19 were 52% in left and 48% in right. Results of vestibular bithermal caloric test and VEMP showed no obvious dysfunction although IV-20 mentioned he experienced vertigo sometimes. Tympanometry results of all participants were completely normal. ABR results of those affected members were consistent with results of PTA, showing moderate to severe sensorineural hearing loss. All affected members failed to pass DPOAE test in most or all frequencies bilaterally. Results of all the unaffected members were normal.

### 3.2. A Novel Nonsense Mutation Was Identified in* POU4F3 *Gene

**Figure 3:**
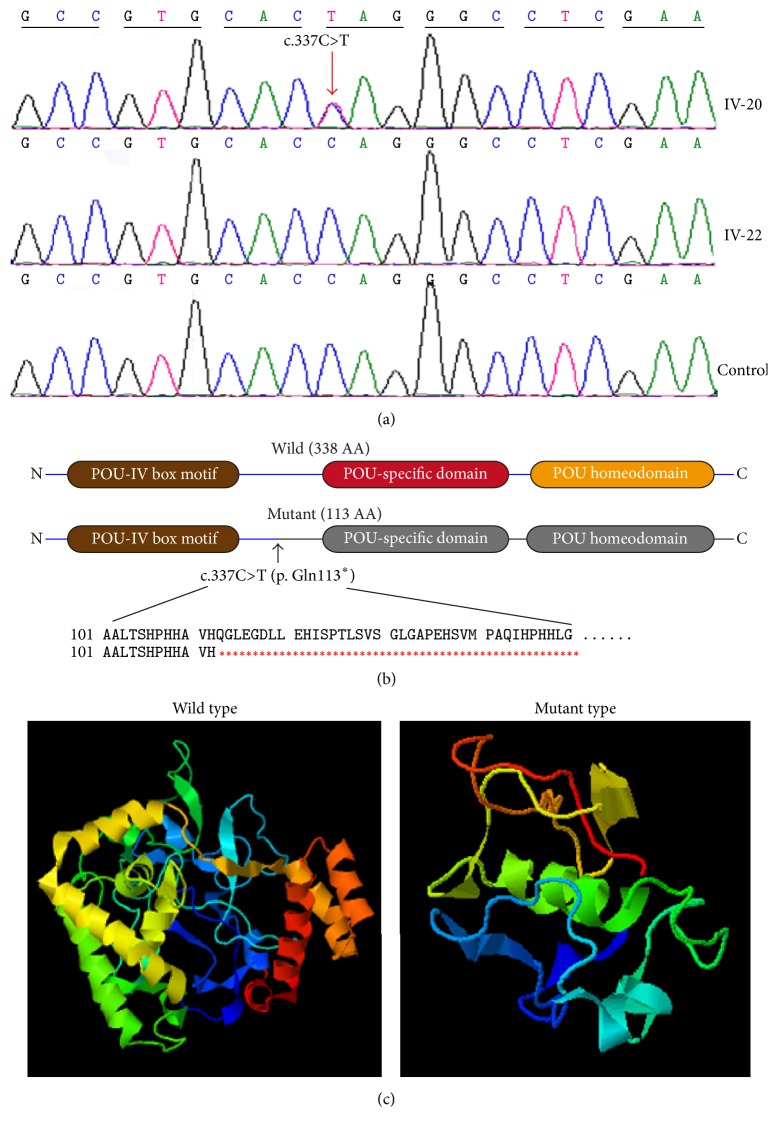
Sanger sequencing confirmation and structural analysis of c.337C>T. (a) Sequencing results of two members in this family and the representative of 200 ethnicity-matched control subjects. Red arrow points to the position of the heterozygous mutation in POU4F3 gene, c.337C>T. (b) The schematic diagram of POU4F3 protein indicating the loss of POU-specific domain and POU homeodomain in mutant protein. (c) Three-dimensional molecular models revealed the incomplete structure of mutant-type protein.

Target region capture sequencing was performed to identify the causative mutation underlying this Chinese family. Single-nucleotide variations were filtered in the dbSNP137, the 1000 Genomes Project, and HapMap8 databases with a 0.5% cutoff of minor allele frequency. Ten variations in nine genes* (POU4F3, OTOF, DSPP, DIAPH1, DFNB31, TPRN, TECTA, TMPRSS3, *and* TRIOBP)* were detected to be possible candidates. By considerations of the autosomal dominant pattern and clinical manifestations in this family, five genes were excluded. Sanger sequencing was performed in all the participating members to confirm the remaining four genes* (DSPP, DIAPH1, TECTA, *and* POU4F3)*. Only one heterozygous nonsense mutation, c.337C>T (p. Gln113^*∗*^), in exon 2 of* POU4F3 *was confirmed (Figure 3(a)). c.337C>T leads to a truncated protein comprising only 112 amino acids (the normal protein contains 338 amino acids) (Figure 3(b)). Among the eight normal-hearing members, c.337C>T was also detected in the proband's little sister (IV-21) who was only six years old. Given that hearing loss caused by mutations of* POU4F3* usually occurs at late age, probably it was still too early for her to present with the symptoms. Sanger sequencing was also conducted in 200 ethnicity-matched control subjects and the mutation was absent in all of them.

Prediction made by Mutation Taster about whether this mutation was pathogenic showed a probability value of 1 (value close to 1 indicates a high “security” of the prediction). A molecular model of* POU4F3* was constructed based on the crystal structure (PDB ID: 1gt0A and 1jvrA) (Figure 3(c)). The constructed model of wild-type protein matched the sequence of* POU4F3* (residues 1–338). The sequence identity between the target and template was 53%, higher than the average 25%. The constructed model of mutant protein matched the target sequence of* POU4F3* (residues 1–112). The sequence identity between the target and template was 25%. We analyzed the wild and mutant structure of* POU4F3 *proteins with Swiss-Pdb Viewer 4.1 software. Compared with the wild-type Pou4f3 structure, the mutant protein structure is incomplete.

### 3.3. Effect of the* POU4F3* Mutation on the Subcellular Localization of Protein

**Figure 4:**
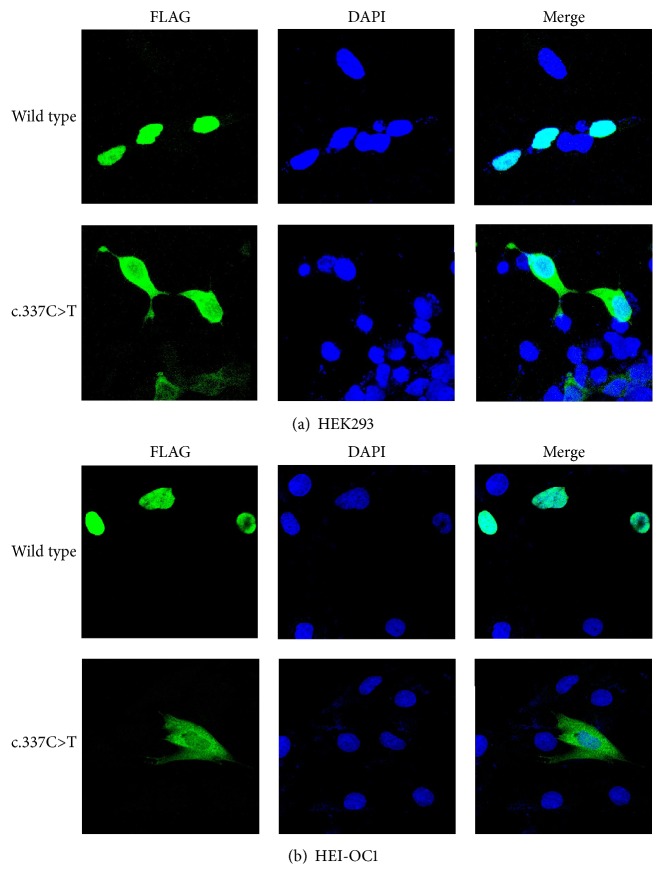
Immunofluorescence analysis after transient transfection in HEK293 and HEI-OC1 cell lines. Images display DAPI in blue, FLAG-tagged protein in green, and merged pictures. (a) In HEK293 cells, mutant protein located mostly in cytoplasm while the wild-type protein was exclusively located in nuclei. (b) Similar results were observed in HEI-OC1 cells.

*POU4F3* is a transcription factor and is exclusively located in the nuclei as previously reported [10, 24]. Subcellular localization is vital for a transcription factor to perform its normal function as it requires the protein to combine with the targets on DNA sequences in nuclei. So the wild-type and mutant-type plasmids were constructed using the pCMV-Tag2B plasmid and cDNA of* POU4F3*. These two constructs were transfected into HEK 293 and HEI-OC1 cell lines, respectively. HEI-OC1 cell line is a conditionally immortalized organ of Corti-derived epithelial cell line [23], which has been shown to be an excellent in vitro system to investigate the cellular and molecular mechanisms involved in ototoxicity and otoprotection of new pharmacological drugs. As the* POU4F3* proteins expressed by these two constructs were fused with N-terminal FLAG-tag, we used anti-FLAG antibody to detect its localization by immunofluorescence analysis under confocal microscopy. Similar results were observed in both cell lines. The normal* POU4F3* protein was exclusively located in the cell nuclei while most of the mutant* POU4F3* protein was located in the cytoplasm. Even though there was still some mutant* POU4F3* protein in the nuclei, the signal was much weaker than that in the cytoplasm (Figure 4).

## 4. Discussion

*POU4F3*, also known as* BRN3C*, is a member of the POU superfamily of transcription factors. Transcription factors bind directly to DNA and regulate the translation of target genes. All 14 members in this superfamily are characterized by comprising two DNA-binding domains, the POU homeodomain, and the POU-specific domain, which are the main functional parts [25].* POU4F3 *protein plays an essential role in the development and maintenance of hair cells in the inner ear sensory epithelia [7]. Targeted null mutation of* POU4F3* resulted in loss of all hair cells in the cochlea and vestibular system of Brn-3c^−/−^ mice and thus led to symptoms of complete hearing loss and severe vestibular dysfunction [6, 26].

In this study, we identified a new nonsense mutation of* POU4F3*, c.337C>T, in a Chinese family which represented progressive hearing loss in an autosomal dominant pattern. This mutation changed the codon CAG to UAG which is a stop codon and thus produces a truncated protein with only 112 amino acids while the normal protein should comprise 338 amino acids. The truncated protein loses the two functional DNA-binding domains, POU-specific domain and POU homeodomain; therefore it might lose its entire function as a transcription factor and result in hair cell apoptosis and progressive hearing loss.

To date, 10 pathogenic variants of* POU4F3* related to DFNA15 have been identified in different countries and ethnicities (Table 1). All the patients were reported to demonstrate the symptoms of postlingual, progressive sensorineural hearing loss and the autosomal dominant pattern of inheritance. The onset age of hearing loss varied a lot from early adult to midlife. All frequencies, especially high frequencies, could be affected resulting in flat to slowly downsloping audiometric configurations in most patients [17]. Interestingly, Brn-3c^−/−^ mice showed severe hearing loss and vestibular dysfunction after birth, while Brn-3c^+/−^ mice showed no auditory and vestibular symptoms [6, 27]. Unlike mice, human would develop late-onset hearing loss when carrying a heterozygous mutation of* POU4F3*. More attention should be paid to* POU4F3* when identifying the cause of a patient with symptoms mentioned above.

No symptoms of vestibular dysfunction were found in the members of this Chinese family. However, the vestibular function in some patients was previously reported to be slightly affected after thorough examinations but the incidence and severity were low [18]. In contrast, distinct vestibular impairment was revealed in Brn-3c^−/−^ mice. The reasons why no obvious vestibular symptoms were found in human are probably that only heterozygous mutations were identified and the normal* POU4F3* allele could produce enough protein to maintain a desirable vestibular function, or functional compensation of vestibular system occurred during the long time span of this disease.

In order to investigate the effect of this novel mutation, we examined the subcellular localization of mutant protein in comparison to a wild-type protein control. The immunofluorescence staining revealed that normal protein was exclusively located in nuclei while mutant protein was located predominantly in cytoplasm in both HEK293 cells and HEI-OC1 cells. Nuclear localization signal (NLS) is an amino acid sequence which plays a key role in guiding transcription factors to cell nucleus and loss of NLS would result in cytoplasmic localization. There are two NLSs in* POU4F3 *according to a previous study [28]. One is monopartite NLS (amino acids 274 to 278), and the other is a bipartite NLS (amino acids 314 to 331). The mutation c.337C>T (p. Gln113^*∗*^) led to the loss of both NLSs which in turn caused the change of localization. Some earlier studies reported that mutant protein produced by transfection could also locate only in nuclei and the difference between normal and mutant protein was the proportion of cells with* POU4F3 *protein outside nuclei [10, 13]. We did not discover this phenomenon after repeated experiments. The possible reason may be these reported missense mutations could not fully destroy the function of NLS.

Identification of targets of* POU4F3* is important for understanding its function and the mechanism of* POU4F3*-related hearing loss. There have been several genes verified to be its downstream targets. Growth factor independence 1 (Gfi1), a zinc-finger transcription factor, was the first-identified target gene of* POU4F3 *and its loss of expression was presumed to be the main cause of outer hair cell degeneration in* POU4F3* mutant individuals [29]. Clough et al. reported that* POU4F3* was capable of activating both BDNF and NT-3 promoters and might be an important regulator of neurotrophic gene expression [30]. Later, Lhx3, a LIM domain transcription factor, was validated to be regulated by* POU4F3* in auditory but not in vestibular system of hair cells [31]. In 2014, the orphan thyroid nuclear receptor Nr2f2 was identified as a new target gene which might be relevant to the survival and development of hair cells [32]. But still, the* POU4F3*-related mechanism of differentiation and maintenance of hair cells is largely unknown.

## 5. Conclusions

In this study we identified a new nonsense mutation c.337C>T in* POU4F3* for the first time in a four-generation Chinese family suffering from autosomal dominant nonsyndromic hearing loss. Functional defects of this truncated protein were revealed by structural analysis and in vitro cellular experiments. Thus far, 10 variants of* POU4F3 *related to DFNA15 have been reported. Mutation of* POU4F3* may not be a rare cause in ADNSHL and routine examination of* POU4F3* is necessary for the genetic diagnosis of hereditary hearing loss in the future.

## Supplementary Material

Summary of 81 Genes related to non-syndromic hearing loss in target region capture sequencing. Name, inheritance pattern, GenBank accession number and exon count of each gene are shown in the table. AR: Autosomal Recessive; AD: Autosomal Dominant; MT: Mitochondrial Inheritance.

## Figures and Tables

**Table 1:** Variants of *POU4F3* related to DFNA 15.

Description	Exon	Amino acid change	Type of variant	Ethnicity	Reference
c.884del8	2	Ile295Thrfs^*∗*^5	Frameshift	Jewish	Vahava et al. (1998) [14]
c.668T>C	2	Leu223Pro	Missense	Dutch	Collin et al. (2008) [10]
c.865C>T	2	Leu289Phe	Missense	Dutch	Collin et al. (2008) [10]
c.662del14	2	Gly221Glufs^*∗*^77	Frameshift	Korean	Lee et al. (2010) [13]
c.694G>A	2	Glu232Lys	Missense	Korean	Baek et al. (2012) [9]
c.977G>A	2	Arg326Lys	Missense	Korean	Kim et al. (2013) [12]
c.603_604delGG	2	Val203Aspfs^*∗*^11	Frameshift	Chinese	Yang et al. (2013) [16]
Deletion of entire gene	2		Deletion	Brazilian	Freitas et al. (2014) [11]
c.491C>G	2	Pro164Arg	Missense	Chinese	Wei et al. (2014) [15]
c.337C>T	2	Gln113^*∗*^	Nonsense	Chinese	This study
