# Erratum to “A Novel Nonsense Mutation of *POU4F3* Gene Causes Autosomal Dominant Hearing Loss”

**DOI:** 10.1155/2017/9202847

**Published:** 2017-06-18

**Authors:** Chi Zhang, Mingming Wang, Yun Xiao, Fengguo Zhang, Yicui Zhou, Jianfeng Li, Qingyin Zheng, Xiaohui Bai, Haibo Wang

**Affiliations:** ^1^Department of Otorhinolaryngology Head and Neck Surgery, Shandong Provincial Hospital Affiliated to Shandong University, Jinan, China; ^2^Shandong Provincial Key Laboratory of Otology, Jinan, China; ^3^Department of Otolaryngology-Head & Neck Surgery, Case Western Reserve University, Cleveland, OH, USA

In the article titled “A Novel Nonsense Mutation of *POU4F3* Gene Causes Autosomal Dominant Hearing Loss” [1], Dr. Xiaohui Bai should be listed as a corresponding author along with Haibo Wang.
