# Supplementary material for: A Novel Nonsense Mutation of POU4F3 Gene Causes Autosomal Dominant Hearing Loss
Source: Neural Plast. 2016 Nov 24;2016:1512831. doi: 10.1155/2016/1512831 (PMC5143711; doi:10.1155/2016/1512831)
Supplement: Supplementary file 1 — Summary of 81 Genes related to non-syndromic hearing loss in target region capture sequencing. Name, inheritance pattern, GenBank accession number and exon count of each gene are shown in the table. AR: Autosomal Recessive; AD: Autosomal Dominant; MT: Mitochondrial Inheritance. [file 1512831.f1.docx]

S1 Table

Summary of 81 Targeted Genes Related to Non-Syndromic Hearing Loss

| Gene | Inheritance | mRNA | Exons |  |
| --- | --- | --- | --- | --- |
| ACTG1 | AD | NM_001199954.1 | 6 |  |
| ATP2B2 | AR | NM_001001331.2 | 23 |  |
| BSND | AR | NM_057176.2 | 4 |  |
| CACNA1D | AR | NM_000720.3 | 49 |  |
| CCDC50 | AD | NM_178335.2 | 12 |  |
| CDH23 | AR | NM_022124.5, | 70 |  |
| CEACAM16 | AD | NM_001039213.3 | 7 |  |
| CLDN14 | AR | NM_144492.2 | 3 |  |
| CLRN1 | AR | NM_174878.2 | 3 |  |
| COCH | AD | NM_001135058.1 | 11 |  |
| CRYM | AD | NM_001888.4 | 10 |  |
| DFNA5 | AD | NM_004403.2 | 10 |  |
| DFNB31 | AR | NM_015404.3 | 12 |  |
| DFNB59/PJVK | AR | NM_001042702.3 | 7 |  |
| DIABLO | AD | NM_019887.5 | 7 |  |
| DIAPH1 | AD | NM_005219.4 | 28 |  |
| DIAPH3 | AD | NM_001258366.1 | 27 |  |
| DSPP | AD | NM_014208.3 | 5 |  |
| ESPN | AR/AD | NM_031475.2 | 13 |  |
| ESRRB | AR | NM_004452.3 | 11 |  |
| EYA4 | AD | NM_004100.4 | 20 |  |
| FOXI1 | AR/AD | NM_012188.4 | 2 |  |
| GIPC3 | AR | NM_133261.2 | 6 |  |
| GJA1 | AR | NM_000165.4 | 2 |  |
| GJB2 | AR/AD | NM_004004.5 | 2 |  |
| GJB3 | AR/AD | NM_024009.2 | 2 |  |
| GJB6 | AR/AD | NM_001110219.2 | 5 |  |
| GPR98 | AR | NM_032119.3 | 90 |  |
| GPSM2 | AR | NM_013296.4 | 15 |  |
| GRHL2 | AD | NM_024915.3 | 16 |  |
| GRXCR1 | AR | NM_001080476.2 | 4 |  |
| HGF | AR | NM_000601.4 | 18 |  |
| ILDR1 | AR | NM_001199799.1 | 8 |  |
| KCNE1 | AR | NM_000219.5 | 4 |  |
| KCNJ10 | AR | NM_002241.4 | 2 |  |
| KCNQ1 | AR | NM_000218.2 | 16 |  |
| KCNQ4 | AD | NM_004700.3 | 14 |  |
| LHFPL5 | AR | NM_182548.3 | 4 |  |
| LOXHD1 | AR | NM_144612.6 | 40 |  |
| LRTOMT | AR | NM_145309.5 | 6 |  |
| MARVELD2 | AR | NM_001038603.2 | 7 |  |
| MIR96 | AD | NR_029512.1 | | |
| MSRB3 | AR | NM_198080.3 | 6 |  |
| MT-RNR1 | MT | — |  |  |
| MT-TS1 | MT | — |  |  |
| MYH14 | AD | NM_001077186.1 | 42 |  |
| MYH9 | AD | NM_002473.5 | 41 |  |
| MYO15A | AR | NM_016239.3 | 66 |  |
| MYO1A | AD | NM_001256041.1 | 29 |  |
| MYO3A | AR | NM_017433.4 | 35 |  |
| MYO6 | AR/AD | NM_004999.3 | 35 |  |
| MYO7A | AR/AD | NM_000260.3 | 49 |  |
| OTOA | AR | NM_144672.3 | 28 |  |
| OTOF | AR | NM_194248.2 | 47 |  |
| OTOG | AR | NM_001277269.1 | 55 |  |
| PCDH15 | AR | NM_001142765.1 | 32 |  |
| PDZD7 | AR | NM_001195263.1 | 17 |  |
| POU3F4 | X-linked | NM_000307.4 | 1 |  |
| POU4F3 | AD | NM_002700.2 | 2 |  |
| PRPS1 | X-linked | NM_002764.3 | 7 |  |
| PTPRQ | AR | NM_001145026.1 | 45 |  |
| RDX | AR | NM_001260492.1 | 16 |  |
| SERPINB6 | AR | NM_001297699.1 | 7 |  |
| SIX1 | AD | NM_005982.3 | 2 |  |
| SLC17A8 | AD | NM_139319.2 | 12 |  |
| SLC26A4 | AR | NM_000441.1 | 21 |  |
| SLC26A5 | AR | NM_198999.2 | 20 |  |
| SMPX | X-linked | NM_014332.2 | 5 |  |
| STRC | AR | NM_153700.2 | 29 |  |
| TECTA | AR/AD | NM_005422.2 | 23 |  |
| TIMM8A | X-linked | NM_004085.3 | 2 |  |
| TJP2 | AD | NM_004817.3 | 23 |  |
| TMC1 | X-linked | NM_138691.2 | 24 |  |
| TMIE | AR | NM_147196.2 | 4 |  |
| TMPRSS3 | AR | NM_024022.2 | 13 |  |
| TPRN | AR | NM_001128228.2 | 4 |  |
| TRIOBP | AR | NM_138632.2 | 8 |  |
| USH1C | AR | NM_005709.3 | 21 |  |
| USH1G | AR | NM_173477.4 | 3 |  |
| USH2A | AR | NM_206933.2 | 72 |  |
| WFS1 | AR/AD | NM_006005.3 | 8 |  |

AR: Autosomal Recessive; AD: Autosomal Dominant; MT: Mitochondrial Inheritance
